# The Essential Role of Sorting Nexin 5 in Virus-Induced Autophagy

**DOI:** 10.3389/fimmu.2022.947384

**Published:** 2022-07-11

**Authors:** Dong-Yi Li, Jun-Hao Wen, Shan Liang, Ji-Xin Tang

**Affiliations:** Guangdong Provincial Key Laboratory of Autophagy and Major Chronic Non-Communicable Diseases, Key Laboratory of Prevention and Management of Chronic Kidney Disease of Zhanjiang, Institute of Nephrology, Affiliated Hospital of Guangdong Medical University, Zhanjiang, China

**Keywords:** sorting nexin 5, autophagy, virus, PtdIns (3)P, endocytosis

## Introduction

Autophagy, a conserved lysosomal degradation pathway, can degrade intracellular waste, such as damaged organelles or misfolded proteins, to meet the metabolic needs of cells and maintain the homeostasis of the intracellular environment (1). In addition, autophagy also plays an essential role in immunity. First, autophagy can be induced by invading virus and provides a way for the host to limit or eliminate the intracellular virus; second, autophagy can also control inflammation by regulating interactions with natural immune signaling pathways, removing endogenous inflammasome agonists, and influencing the secretion of immune mediators; third, autophagy involves in antigen presentation and T polarization (2, 3).

The virus typically enters host cells *via* endocytosis and thus remains enclosed in the endosomes during the early stages of infection, making it difficult to identify by the host cellular autophagy. There is unknown how the virus can be specifically identified as selective autophagy cargo so as to be eliminated by host cellular autophagy and how does virus-induced autophagy occur in mammalian cells. Recently, Dong et al. found that the endosomal protein sorting nexin 5 (SNX5) is critically required for virus-induced, but not for other factor induced autophagy (4).

## SNX5: A Protein Involved in Endosomes

As a member of the sorting nexin family, SNX5associates with the retromer, a complex of proteins that has been shown to be important in recycling transmembrane receptors from endosomes to the trans-Golgi network, and plays an important role in endosomal transport and protein sorting. SNX5 mainly contains two major domains: Phox homology (PX) domain and carboxy-terminal Bin, Amphiphysin, Rvs (BAR) domain, both of which are evolutionarily conserved domains. PX domain mainly recognizes the sorting motif of cargo (proteins to be sorted), and can help SNX5 to localize to the endosomal membrane of endocytosis pathway (5). SNX5-BAR domain mediates the biogenesis of cargo transport carriers and plays an essential role to sense and drive membrane bending (6, 7). SNX 5 is involved in endosomal sorting and is also related to PI signal pathway. SNX5 participates in caspase 9 mediated IGF2R retrieval mechanism (8) and forms a recycler complex with SNX4 and SNX17, which mediates the autophagosomal components recycling (ACR) process and helps the autophagosome membrane components on autophagy lysosomes to be recycled (9). In addition, SNX5 is also involved in the progression of cancers such as hepatocellular carcinoma (10) head and neck squamous cell carcinoma (11) and clear cell renal carcinoma (12).

Most of the virus, such as hepatitis B virus (13), SARS-COV-2 (14), influenza virus (15), and herpes virus (16)infect host cells *via* an endocytic vesicle. Only a small number of viruses infect host cells by means of genetic material injection, such as phages, and the targeted cells are generally cells with the cell wall and outer membrane (17). Recently, it has been found that SNX5 is involved in the virus-induced autophagy. SNX5 can inhibit virus replication in an autophagy-dependent manner and reduce the susceptibility and lethality of host cells (4).

## SNX5 in Virus-Induced Autophagy

**Figure 1:**
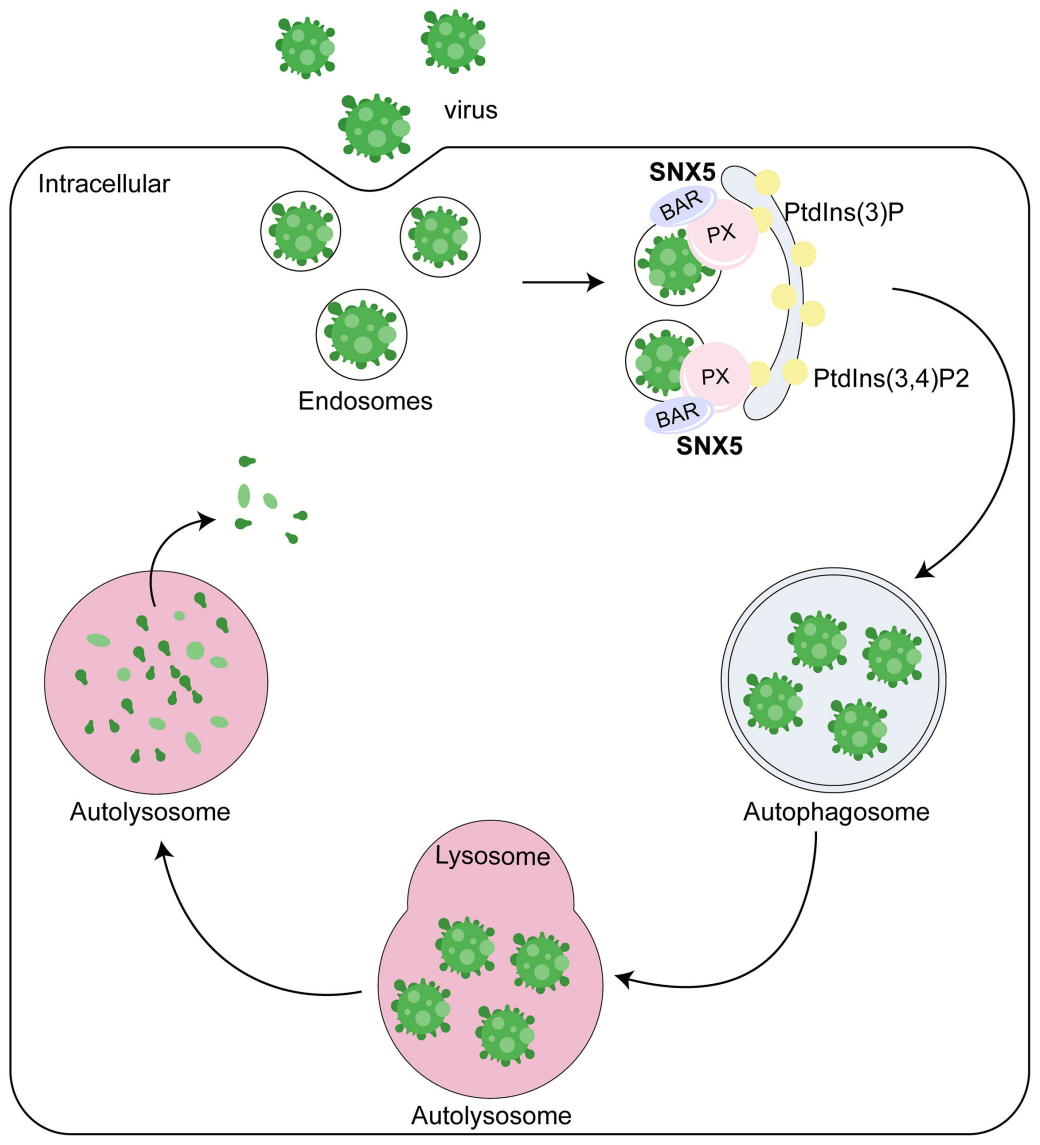
Role of SNX5 in the antiviral process. SNX5 mainly contains two major domains: phox (PX) domain and BAR domain. PX domain mainly recognizes the sorting motif of cargo and binds to phosphatidylinositol-3-phosphate (PtdIns (3)P) or PtdIns (3,4)P2 to regulate the localization of SNX5 to the autophagosome membrane. SNX5-BAR domain mediates the biogenesis of cargo transport carriers and plays an essential role to sense and drive membrane bending. Therefore, SNX5 can promote the virus’s clearance, reduce the virus’s susceptibility and lethality to host cells and inhibit virus replication in an autophagy-dependent manner.

A team led by the late Beth Levine found that SNX5 is critically required for virus-induced, but not for basal or stress-induced autophagy through genome-wide short interfering RNA screens (4) (**Figure 1**). Sindbis virus (SIN) and a genetically engineered strain of herpes simplex virus type 1 (HSV-1) lacking the beclin 1-binding domain (BBD) of the HSV-1 neurovirulence protein ICP34.5 that cannot inhibit host autophagy (HSV-1ΔBBD) failed to induce autophagy after the knockout of SNX5 *via* CRISPR-Cas9, but this defect can be rescued by expressing the recombinant wild-type SNX5. These results suggest that SNX5 may be necessary for most virus-induced autophagy. Besides, they also found that SNX5 only had an effect on virus-induced autophagy, but had no effect on basal autophagy, starvation or mTOR-induced autophagy and bacterial heterophagy. Therefore, SNX5 is specifically required for virus-induced autophagy, and the role of SNX5 in virus-induced autophagy is unlikely to be related to its reverse transport function or other endocytosis function.

*In vivo* experiments showed that neonatal Snx5 knockout (Snx5-/-) mice were more susceptible to lethal infection with SIN, Chikungunya virus (CHIKV), or West Nile virus (WNV) virus than their wild-type littermates (Snx5+/+) (4). Whereas, there has no difference in mortality between Snx5+/+ and Snx5−/− mice when infection with autophagy-suppressive viral strains such as SIN or HSV-1ΔBBD, suggesting that SNX5 also plays an important antiviral defense function through autophagy *in vivo* (4).

Mechanistically, SNX5 can be selectively recruited to the endosomes containing virus particles, sensing membrane curvature, driving membrane bending and increasing membrane curvature through its BAR domain (**Figure 1**). The key mediator regulating the generation of PtdIns (3)P is ATG14-containing class III phosphatidylinositol-3-kinase complex 1 (PI3KC3-C1), which contains a component molecule ATG that can sense membrane curvature. With the change of membrane curvature, the activity of PI3KC3-C1 kinase and the efficiency of PtdIns (3)P production changed. Generally speaking, the greater the membrane curvature, the higher the activity of PI3KC3-C1 kinase and the higher the PtdIns (3)P generation efficiency. SNX5 can directly interact with PI3K complex 1 and regulate it through modulating membrane curvature on the low-curvature membrane structures, such as endosomes. By doing this, SNX5 can further promote the biogenesis of autophagy, and autophagosome formation by improving the activity of PI3KC3-C1 kinase and increasing PtdIns (3)P production. Besides, SNX5 can initiate the clearance of virus, reduce the susceptibility and lethality of virus to host cells. And it can inhibit virus replication in an autophagy dependent manner. The ability of SNX5 to change membrane curvature and PI3KC3-C1 kinase activity plays an essential role in virus-induced autophagy (4).

## Discussion

Although autophagy plays an essential role in fighting virus infections and eliminating viruses. But, in some cases, the existence of autophagy may have a negative effect on the antiviral response of cells and even enhance the replication of some viruses. Therefore, SNX5 itself, which is necessary for virus-induced autophagy, is likely to be an accomplice of the virus in some cases. Besides, the endosomal membrane wrapping virus particles is also an accomplice to help the virus to escape host cell attack.

After the viruses entered into the lumen of endosomes or the endoplasmic reticulum (ER), they will get some cues, such as exposure to low pH or proteolytic cleavage, which will trigger changes in the virus particle, and the activated viruses can penetrate the vacuolar membrane, enter the cytoplasm or nucleus to replicate their genetic material, synthesize virus protein, and so on (17). The cytoplasmic retinoic acid-inducible gene I (RIG-I) receptor is activated after recognizing viral RNA and increases the expression of antiviral genes and antiviral activities such as type I interferons and inflammatory cytokine genes through cascaded reaction with downstream molecules. However, SNX5 can negatively regulate RIG-I-like receptor (RLR)-mediated antiviral signaling by inhibiting virus-induced RIG-I receptor expression and weakening the interaction between downstream molecule virus-induced signaling adaptor (VISA) and Tumor necrosis factor (TNF) receptor-associated factor 2/5 (TRAF2/5). Overexpression of SNX5 can inhibit the virus-induced activation of nuclear factor kB (NF-kB) and Type-I interferons (IFN) regulatory factor 3 (IRF3), which may lead to a decrease in the cell’s ability to fight viral infection (18).

Although, SNX5 is essential for virus-induced autophagy and can inhibit the transmission of the virus and reduce the harm of the virus to the host. It may be a double-edged sword for the hosts, considering that some viruses may kidnap the host’s autophagy process to serve its replication and transmission. Therefore, we should take this into account when we want to target SNX5 to against the virus. Our understanding of SNX5 in virus-induced autophagy is far from enough. For example, how does SNX5 only recognize endosomes that contain virus particles rather than other substances? In the future, it will be interesting to investigate how SNX5 can specifically “recognizes” virus-infected endosomes and promote the activity of autophagy. This will be an important step towards understanding how specificity is achieved in selective autophagy and how can we use this to against and to combat the spread of the virus, thereby reducing its damage to the host.

## Author Contributions

D-YL, J-HW, SL, and J-XT wrote the first draft of the manuscript. All authors contributed to the article and approved the submitted version.

## Funding

This work was supported by Natural Science Foundation of Guangdong Province (2019A1515110152) and Discipline construction project of Guangdong Medical University (4SG21229G).

## Conflict of Interest

The authors declare that the research was conducted in the absence of any commercial or financial relationships that could be construed as a potential conflict of interest.

## Publisher’s Note

All claims expressed in this article are solely those of the authors and do not necessarily represent those of their affiliated organizations, or those of the publisher, the editors and the reviewers. Any product that may be evaluated in this article, or claim that may be made by its manufacturer, is not guaranteed or endorsed by the publisher.
